# Where’s *Waldo*? A new commensal species, *Waldo arthuri* (Mollusca, Bivalvia, Galeommatidae), from the Northeastern Pacific Ocean

**DOI:** 10.3897/zookeys.316.4256

**Published:** 2013-07-12

**Authors:** Paul Valentich-Scott, Diarmaid Ó Foighil, Jingchun Li

**Affiliations:** 1 Santa Barbara Museum of Natural History, 2559 Puesta del Sol Road, Santa Barbara, California 93105 USA; 2 Museum of Zoology, Department of Ecology and Evolutionary Biology, University of Michigan, 1109 Geddes Avenue, Ann Arbor, Michigan 48109-1079 USA

**Keywords:** Commensal relationships, Bivalvia, Galeommatidae, *Waldo*, Echinodea, urchin, taxonomy

## Abstract

A galeommatid bivalve mollusk, representing a new species, is described from off the coasts of California and Vancouver Island, British Columbia. The new bivalve has a commensal relationship with the heart urchin, *Brisaster latifrons*. It has been observed crawling between the oral spines of this urchin, frequently near the peristome. The bivalve has been recorded from 80 (Vancouver Island) to 444 (southern California) meters depth, in muddy sediments.

In common with other galeommatoideans, the new species broods its young; however it differs from the large majority of commensal members in lacking planktotrophic larval development.

*Waldo arthuri*, new species, has multiple morphological, ecological and developmental similarities to other members of the genus *Waldo* Nicol, 1966, from the southern Atlantic and Antarctic Oceans. This is most pronounced for the Argentine species, *Waldo paucitentaculatus* Zelaya & Ituarte, 2013, *Waldo arthuri*’s sister speciesin nuclear and mitochondrial gene trees. Despite this close relationship, *Waldo arthuri* is phylogentically distinct and possesses several hinge, shell sculpture, foot, and mantle tentacle characteristics that merit its description as new.

## Introduction

The unusual lifestyles of galeommatoidean bivalve mollusks have been extensively studied for over 185 years (Turton 1825). They are found in all oceans, occupy benthic habitats from the intertidal to continental shelf depths, and comprise large numbers of both free-living and commensal species. A spectrum of commensal relationships has been documented involving diverse invertebrate hosts including echinoderms, crustaceans, and annelids (Boss 1965, Chavan 1960, 1969, Dall 1884, 1899, Gage 1966, 1979, Goto et al. 2012, Morton and Scott 1989). This commensal lifestyle is robustly correlated with living in soft sediments, and the evolution of biotic associations with infaunal bioturbating hosts may have been a prerequisite for the diversification of Galeommatoidea in soft-bottom benthos (Li et al. 2012).

An undescribed galeommatid species was discovered in the late 1980’s in two regions of the northeastern Pacific Ocean: Vancouver Island, British Columbia and Santa Barbara, California. The new species lives commensally with the heart urchin, *Brisaster latifrons* (Agassiz, 1898) and is morphologically distinct from other known irregular echinoid commensals (Coan et al. 2000, Coan and Valentich-Scott 2012, Gage 1966, Morton and Scott 1989, Oliver 2012, Ponder 1967, 1971, Yamamoto and Habe 1974, Zelaya and Ituarte 2002, 2013).

Coan et al. (2000) provisionally identified the specimens as “*Divariscintilla*” sp. A, but this generic placement was thrown in doubt after Zelaya and Ituarte’s (2002) redescription of a very similar species, *Waldo parasiticus* (Dall, 1876), the type species of genus *Waldo*. Moreover, Zelaya and Ituarte (2002) described a new congener in the Southern Ocean, *Waldo trapezialis*, which is also lives on the spines of irregular echinoids and is morphologically similar to the new northeastern Pacific species. Two additional new South Atlantic *Waldo* species have recently been discovered (Zelaya and Ituarte 2013) and specimens were kindly forwarded to us for genotyping and morphologic examination. This, together with the receipt of fresh specimens from British Columbia, has prompted us to formally describe this species and test its phylogenetic relationships with South Atlantic *Waldo* species.

## Materials, methods, abbreviations

Specimens of the heart urchin *Brisaster latifrons* were dredged in Barkley Sound, British Columbia, Canada by invertebrate biology classes held at the Bamfield Marine Sciences Centre on two occasions: in June 1989 from off Sandford Island at 80 m depth (48°51.47'N, 125°08.95'W), and in August 2011 from subtidal depths in the Imperial Eagle Channel (48°55.052'N, 125°13.657'W). On both occasions, live specimens of *Waldo* were observed attached to the ventral surface of the urchins. The bivalves were removed from their urchin hosts for study using dissecting microscopy and scanning electron microscopy and some were preserved in 95% ethyl alcohol for molecular characterization.

In 1986, independent sampling via box corer off Santa Barbara, California yielded additional specimens of the new species. It has subsequently been collected off Monterey Bay, Point San Luis, Los Angeles, and San Diego, California. None of the California *Waldo* specimens were directly collected from a host, but in several instances *Brisaster latifrons* was also found in the same samples. All California specimens were preserved in 4% formalin and then transferred to 70% ethyl alcohol.

For the molecular phylogeny, specimens of *Waldo arthuri*, collected in British Columbia in 2011, were genotyped together with specimens from two recently discovered species of *Waldo* from Puerto Deseado, Argentina: *Waldo digitatus* Zelaya & Ituarte, 2013, and *Waldo paucitentaculatus* Zelaya & Ituarte, 2013. Two *Lasaea* lineages were used as outgroups: *Lasaea australis* (from Esperance, Australia) and an unidentified direct-developing *Lasaea* sp. (from Hong Kong). Ethanol-preserved voucher material of the genotyped *Waldo* and *Lasaea* species have been deposited into the Museum of Zoology, University of Michigan (UMMZ 203919, 203927, 203928).

### DNA amplification

A small piece of mantle tissue from each specimen was isolated for genomic DNA extraction using the Omega Biotek E.Z.N.A. Mollusc DNA Kit (Omega. tech). Fragments of two ribosomal genes, the mitochondrial large subunit 16S and the nuclear large subunit 28S, were used to reconstruct the phylogenetic relationships of the North American and Argentine *Waldo* taxa. For all species except *Waldo paucitentaculatus*, the 16S gene fragment was amplified using the *Lasaea* spp.primer set 16SLasF (5’-TAGATTAAGGGTTGGGCCTG-3’)/16SLasR (5’-GCCTAAATGGTAAGACTGTTCG-3’) (Li et al. unpublished data) following a touchdown PCR protocol. The initial annealing temperature (55°C) was decreased by 2°C per cycle until the final annealing temperature (48°C) was reached, then the reaction was continued for an additional 35 cycles. Because the target gene of *Waldo paucitentaculatus* failed to amplify with this protocol, an internal, *Waldo* specific primer set was developed and a doubly-nested amplification procedure was adopted to improve the PCR process. The first round of PCR was performed as above using the 16SLasF/16SLasR primer set. Products from the first PCR were then used as templates for a second round touchdown PCR using the newly developed internal primers 16SWaldoF (5’-GGCCTGCCCGGTGATAA-3’)/16SWaldoR1 (5’-CAACATCGAGGTCGCAAAC-3’). The target 28S fragment for all species was amplified using the primer combination D23FLas (5’-CCGCATAGAGGCAAACGGGT-3’) (Li et al. 2013)/D6R (5’-CGAAGTTTCCCTCAGGATAGCTGG-3’) (Park and Ó Foighil 2000), following a standard PCR protocol with an annealing temperature at 50°C. All PCR products were sequenced at the University of Michigan Sequencing Core facility and all *Waldo* spp. sequences were deposited in GenBank under the accession numbers JX646678-JX646693.

### Phylogenetic analyses

The 16S and 28S sequences were aligned respectively using ClustalW (Thompson et al. 1994) implemented in CodonCode Aligner 3.1.7 (CodonCode Corporation), and corrected by eye. The 16S gene segments amplified using the *Waldo* specific primers (339 nt) were shorter than the ones using 16SLasF/16SLasR primers (394 nt). Thus the homologous 339 nt fragment was used for further analyses. The 28S gene segment has a length of 707 nt.

Bayesian and maximum likelihood (ML) inferences were used to reconstruct the *Waldo* phylogeny for both genes fragments. For each dataset, the appropriate substitution model was selected by JModelTest 2.0.2 (Guindon and Gascuel 2003; Darriba et al. 2012) using the Akaike information criterion. Bayesian analysis was performed using MrBayes 3.1.2 (Huelsenbeck and Ronquist 2001). The Markov chain Monte Carlo (MCMC) was run for 1 million iterations with trees sampled every 1000 iterations. Two independent runs were performed with two cold and two heated chains in each run. The cumulative split frequencies were observed to be below 0.01 and all parameters were examined in Tracer 1.5 (Rambaut and Drummond 2009) to ensure convergence and proper mixing. The first 250 trees were burned in according to the convergence diagnosis and a 50% majority consensus tree was obtained. Maximum likelihood analyses were conducted with 100 bootstrap replicates using the RAxML (Randomized Axelerated Maximum Likelihood) 7.2.8 (Stamatakis 2006; Stamatakis et al. 2008) online serves hosted at the Vital-IT (http://www.vital-it.ch) Center for high-performance computing of the SIB Swiss Institute of Bioinformatics. The best-scoring trees from the analyses were obtained to represent the phylogeny.

**Abbreviations:** SBMNHSanta Barbara Museum of Natural History, Santa Barbara, California, USA; UMMZUniversity of Michigan, Museum of Zoology, Ann Arbor, Michigan, USA.

## Systematic account

### Superfamily Galeommatoidea Gray, 1840
Family Galeommatidae Gray, 1840

#### Waldo

Nicol, 1966

http://species-id.net/wiki/Waldo

Waldo Nicol 1966. Type species (original designation) *Lepton parasiticus* Dall, 1876. Recent, Antarctica.

##### Description.

Shell small (length less than 5 mm), ovate to trapezoidal, extremely thin, fragile, translucent to opaque, gaping ventrally and on anterior and posterior ends; sculpture of commarginal striae, weak radial ribs in some; periostracum thin to thick, translucent to white; hinge plate narrow, adults edentate; ligament internal; mantle papillate, reflected, covering most of outer shell surface; long, slender mantle tentacles extend well past shell margin; foot elongate, thin, triangular to cylindrical, heel strong to absent; with one demibranch on each side.

##### Discussion.

Zelaya and Ituarte (2002) revived the use and understanding of this genus, with the redescription of the type species, *Waldo parasiticus*, and the description of a new species: *Waldo trapezialis*. They described, for the first time, the gross anatomy of members of the genus and suggested a possible position within the Galeommatoidea. All species are likely to be obligate commensals with echinoid echinoderms. Two additional species were described from the southwestern Atlantic Ocean (Zelaya and Ituarte 2013).

#### Waldo arthuri

Valentich-Scott, Ó Foighil & Li sp. n.

urn:lsid:zoobank.org:act:1000CA2C-56A1-4846-B8B0-91D3AD0199CE

http://species-id.net/wiki/Waldo_arthuri

Figures 1A–H , 2A–C

Divariscintilla “ ” sp. A Coan et al. 2000: 314

##### Description.

**Figure 1.**
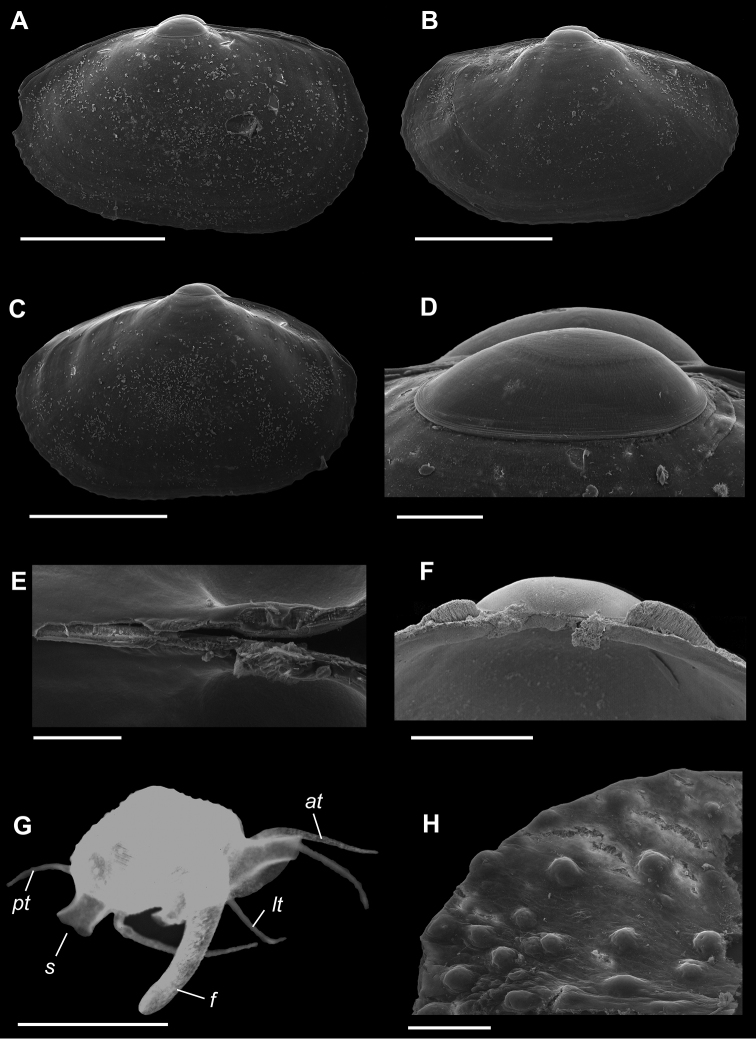
**A–H** *Waldo arthuri* new species **A–E** paratypes, SBMNH 149934 **A–C** Exterior of left valve **D** Prodissoconch **E** Close up of hinge of both valves **F** Close up of hinge of right valve **G** Live animal with extended mantle and mantle tentacles; posterior mantle tentacle (*pt*); siphon (*s*), foot (*f*), lateral mantle tentacle (*lt*), anterior mantle tentacle (*at*) **H** Detail of mantle papillae. **A–C, G** scale bar = 1 mm; **D–F, H** scale bar = 100 µm.

**Shell** extremely thin, fragile, moderately inflated, translucent; equilateral to slightly longer posteriorly, anterior end slightly flared to gently sloping (Figure 1A-C); shell margins only weakly gaping if at all. **Prodissoconch** non-umbonate, D-shaped, with a greatly reduced PII comprised of a small number of faint commarginal striae bordering the metamorphic prodissoconch/dissoconch boundary (Figure 1D), prodissoconch length ranged from 338 to 357 µm (n=8) (Figure 1B). **Dissoconch sculpture** of commarginal striae, plus low broad irregular radial ribs; external sculpture variable, radial ribs absent to moderately strong, especially on anterior and posterior ends in some specimens. Beaks low, wide. **Hinge plate** extremely narrow, edentulous (Figures 1E, F). Length to 5 mm.

**Mantle** large, reflected, covering approximately 80% of outer shell surface when fully extended, not covering umbones (Figure 1G); mantle can be completely retracted into the shell; reflected portion papillate (Figure 1H); fused posteroventrally; facultative exhalant siphon, trumpet-shaped, non-papillate; anterior end thin, non-papillate.

**Mantle tentacles** long, extend well past shell margins (Figure 1G). Adult with projecting anterior pair, two laterally projecting pairs just posterior to anterior tentacles (one pair on each side); lateral tentacles not present on individuals less than 1 mm in length; ventral pair of tentacles just anterior of exhalant siphon (largest of all tentacles, in adults up to length of shell); single posterior tentacle projects dorsally to the exhalant opening. When animals are actively crawling, it appears that the tentacles might be used as levers to navigate between the urchin spines.

**Foot** large, exceeds the length of the shell when fully extended, vermiform, without heel (Figure 1G); long ventral byssal groove extending to end of smooth foot tip. This species is an active crawler, and can also attach to the host by byssal threads.

**Ctenidia** with one demibranch on each side, comprised of about 12-15 widely spaced filaments in larger specimens.

##### Development.

**Figure 2.**
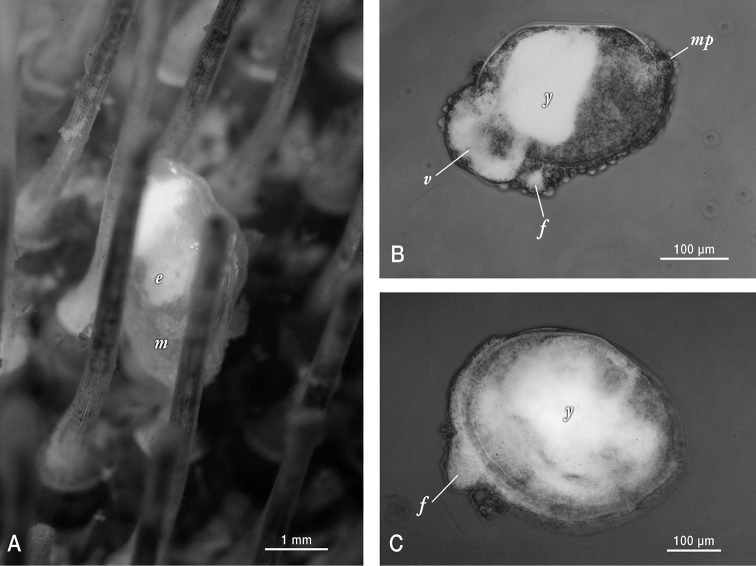
Photographs of live *Waldo arthuri* material sampled in Barkeley Sound in 1989. **A** Brooding adult attached to its host. Note the papillated mantle (*m*) that is partially retracted and the presence of ~ 200 µm diameter white yolky early embryos (*e*) in its ctenidia, visible through the transparent shell **B** Micrograph of mid-late development embryo (equivalent to the pediveliger stage in pelagic developing bivalves) that was dissected from its brooding parent’s ctenidia. Labels indicate protruding foot (*f*), modified non-ciliated velum (*v*) with partially consumed yolk reserves (white areas) and mantle papillae (*mp*) in addition to a dense mass of yolk (y) sequestered in the anterior shelled half of the embryo **C** Micrograph of smallest/youngest (20 µm of dissoconch growth) specimen observed attached to an urchin host. Note the protruding foot (*f*) and the apparent presence of persistent yolk reserves (*y*) dispersed throughout much of the juvenile’s visceral mass.

The reproduction is typical of galeommatoideans, in that the animal is hermaphroditic, and the young are brooded in the ctenidia. Two brooding individuals sampled in 1989 showed early and mid developmental stages respectively. Fecundity was low; the early developmental stage individual (3.8 mm length) had 160 yolky embryos all at the blastula stage (approximately 200 µm in diameter) (Figure 2A). The second specimen was brooding mid-late stage shelled embryos (~ 270 µm length) with a protruding unciliated velum containing partially depleted yolk reserves, a larger dense mass of yolk present in anterior visceral mass, a papillate mantle that extended outside of the valve margins, and a protruding foot. The smallest non-brooded individual observed (370 µm length) byssally attached to its urchin host, had attained a modest (20 µm) increment of dissoconch growth, but notably still had visible yolk reserves dispersed across its visceral mass (Figure 2C). Although we have not observed early ontogeny, these characteristics, together with the non-umbonate prodissonch, point unambiguously toward a non-pelagic developmental mode.

##### Type locality.

USA, California, San Luis Obispo County, off Pt. San Luis; 35°05'18'N, 121°00'54"W; 409 m.

##### Type material.

Holotype, SBMNH 235142, conjoined shell and anatomy, length 2.5 mm, height 1.5 mm. Holotype comprises two conjoined valves, with anatomy, preserved in 70% ethyl alcohol. Given its wet preservation and small size we were unable to capture high quality photographs of the holotype.

7 Paratypes, SBMNH 149934, same locality as holotype (Figures 1A–E), specimens mounted on SEM stub; Figure 1A length 2.45 mm, height 1.45 mm; Figure 1B length 2.55 mm, height 1.45 mm; Figure 1C length 2.61 mm, height 1.63 mm.

3 Paratypes, SBMNH 235142, same locality as holotype (preserved in 100% EtOH).

4 Paratypes, SBMNH 149933, Canada, British Columbia, Sanford Island, Barkley Sound; 48°51'28"N, 125°08'57"W; 80 m, attached to *Brisaster latifrons*.

34 Paratypes, UMMZ 303919, Canada, British Columbia, Imperial Eagle Channel; 48°55.052'N, 125°13.657'W (preserved in 100% EtOH).

##### Distribution and habitat.

Canada, British Columbia, Barkley Sound, Sanford Island, 80 meters, and Imperial Eagle Channel in soft sediments; and United States, California, from Monterey Bay to La Jolla, from 113 to 444 meters [SBMNH].

Ten juvenile specimens from the intertidal zone of Smeaton Bay, Alaska (55.4°N, 130.6°W) [SBMNH 149330] are too small to be identified to species, but might also be *Waldo arthuri*.

##### Commensal relationship.

Crawling on the oral surface of the heart urchin *Brisaster latifrons*, primarily near the peristome. In 1989, most Barkley Sound heart urchins examined had a single bivalve although up to 3 specimens were collected on a single host. In 2011, the commensals were more plentiful: 22/33 urchins bore at least 1 commensal (mean = 2.7 clams/urchin); the maximum number on an individual host was 23 clams.

##### Discovery.

Independently discovered in the late 1980’s by Arthur Fontaine and Diarmaid Ó Foighil in British Columbia and Paul Valentich-Scott and Donald Cadien in southern California.

##### Etymology.

This species is named after Dr. Arthur Fontaine, Professor Emeritus of Biology at the University of Victoria, British Columbia, Canada.

##### Comparisons.

Table 1 provides characteristics to separate *Waldo arthuri* from other members of the genus. The Antarctic *Waldo parasiticus* is subequilateral, has a distinct anterior gape, and lacks the elongate anterior and posterior tentacles. *Waldo trapezialis*, has a strong saddle shaped internal ligament, is subequilateral, and lacks strong radial sculpture. *Waldo digitatus* Zelaya & Ituarte, 2013 lacks the radial sculpture and has a large number of mantle tentacles ventrally. *Waldo arthuri* is closest to *Waldo paucitentaculatus* Zelaya & Ituarte, 2013, which has wider, stronger radial ribs, a strongly crenulate ventral margin, and a much narrower anterior end.

**Table 1.** Comparison of morphologic characteristics of members of the genus *Waldo*. <br/>

**Taxa**	**Shell shape**	**Living animal**	**Pedal mantle tentacles**	**Crenulate ventral margin**
*Waldo arthuri*	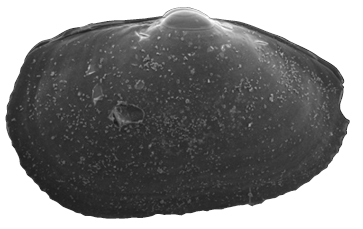	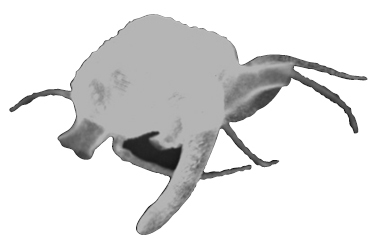	1 pair	no
*Waldo parasiticus*	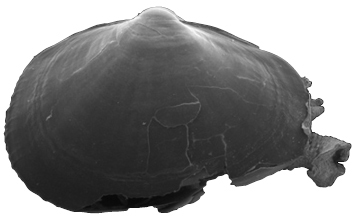	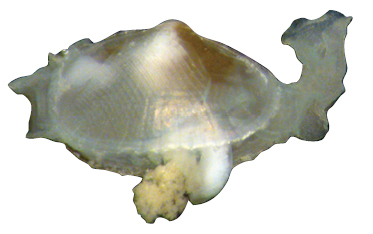	5 pair	yes
*Waldo trapezialis*	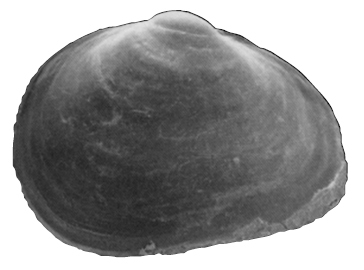	unfigured	3 pair	no
*Waldo paucitentaculatus*	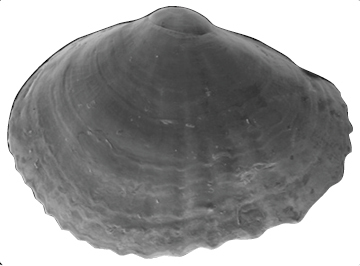	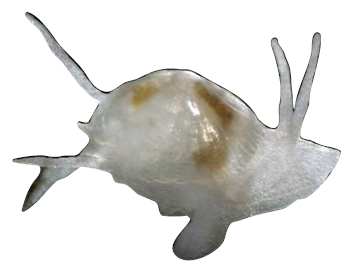	1-3 pair	yes
*Waldo digitatus*	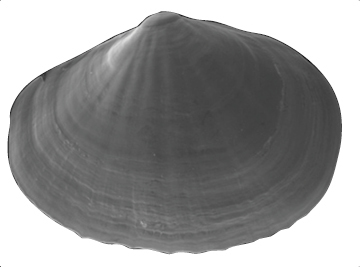	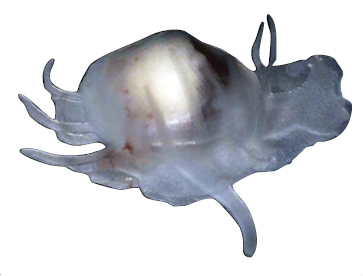	5-15 pair	slightly

*Scintillona bellerophon* Ó Foighil & Gibson, 1984 is the only other galeommatid from the northeast Pacific that has been recorded as an epibiont on echinoderms. This species attaches externally to the sea cucumber, *Leptosynapta clarki* (Heding, 1928). *Scintillona bellerophon* has cardinal teeth in both valves. The shell is much thicker, and not transparent, when compared with *Waldo arthuri*.

A species from Japan and Hawaii, *Scintillona stigmatica* (Pilsbry, 1921), has been collected on the heart urchin, *Brissus latecarinatus* (Leske, 1778). Yamamoto and Habe (1974) illustrate this bivalve on the ventral surface of the urchin, in an arrangement very similar to *Waldo arthuri*. However *Scintillona stigmatica*, as with *Scintillona bellerophon*, has a cardinal tooth in each valve. In addition, *Scintillona stigmatica* has a red-brown stripe of color running laterally from the umbones to the posteroventral margin.

In the eastern Atlantic Ocean, Gage (1966) documented two species of “*Montacuta*” attached to spantangoid urchins. Both species have a dentate hinge, and are easily separated from the new species.

Other similar North American species include those belonging to *Divariscintilla*. Mikkelsen and Bieler (1989, 1992) describe five species of *Divariscintilla* from Florida. Externally these species all have a papillate, reflected mantle, and long mantle tentacles, similar to the new species. However members of *Divariscintilla* have distinct cardinal teeth.

## Molecular analysis

**Figure 3.**
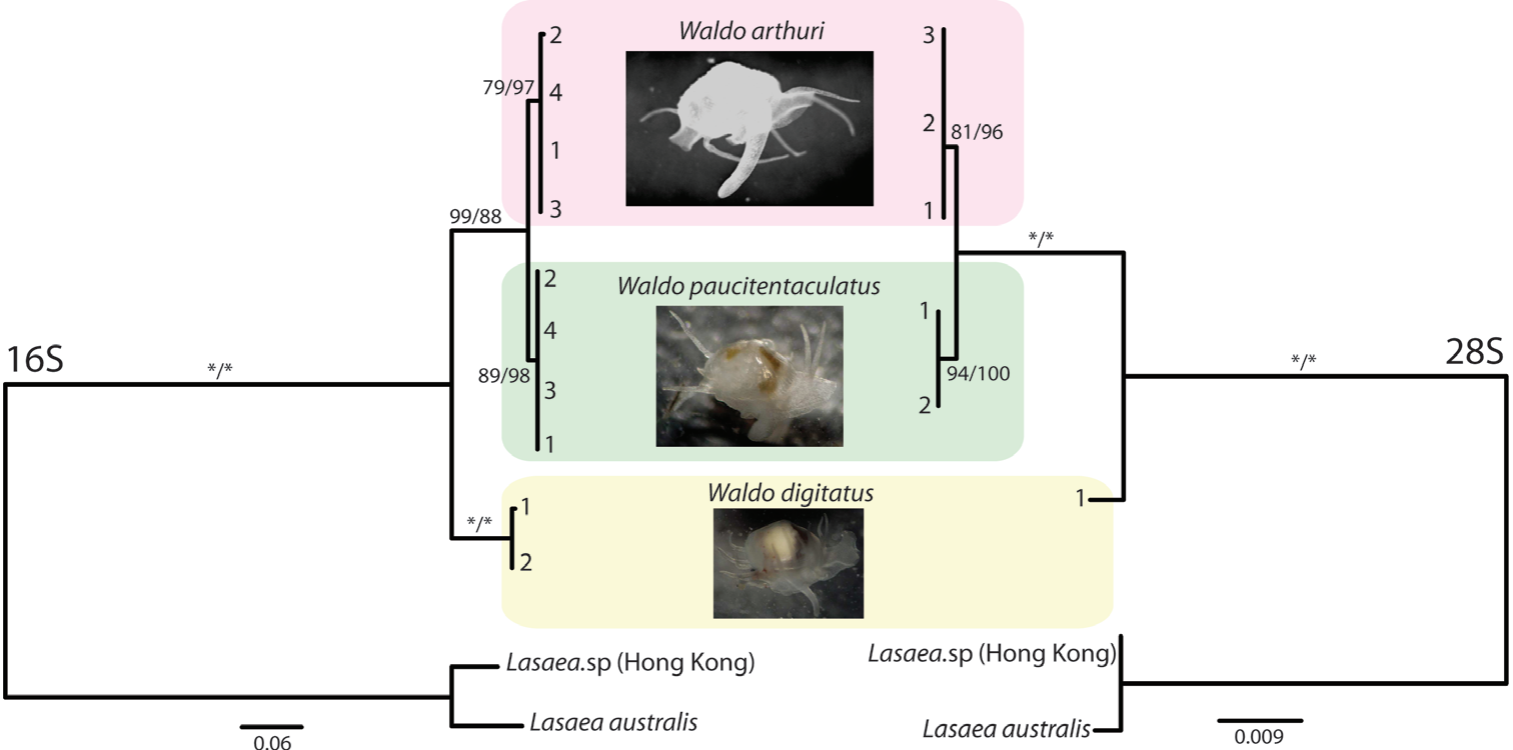
16S and 28S phylogenies of three *Waldo* species. Numbers at branch tips represent specimen ID numbers. Support values along branches are reported as Bayesian posterior probabilities and ML bootstrap values respectively. An asterisk indicates a support value of 100. The scale bars represent numbers of substitutions per site.

Results from the phylogenetic analyses are shown in Figure 3. For the mitochondrial 16S gene, we successfully amplified sequences from four individuals each of *Waldo arthuri* and *Waldo paucitentaculatus*, and two sequences from *Waldo digitatus*. Both Bayesian and ML analyses gave congruent topologies. All three species of *Waldo* formed their own monophyletic clade with relatively high statistical support. *Waldo arthuri* nested among the two South Atlantic congeners, placing robustly sister to *Waldo paucitentaculatus* (Figure 3) and thereby rendering *Waldo digitatus* basal. The mean genetic distances among the three species for the mt 16S rDNA gene fragment were: 2.2% (*Waldo arthuri*/ *Waldo paucitentaculatus*), 11.6% (*Waldo digitatus* / *Waldo paucitentaculatus*) and 12.4% (*Waldo digitatus* / *Waldo arthuri*). Mean genetic distance within *Waldo digitatus* is very modest (0.3%). No intraspecific genetic variation was detected for *Waldo arthuri* and *Waldo paucitentaculatus*.

Specimens of *Waldo arthuri* (N=3), *Waldo paucitentaculatus* (N=2)and *Waldo digitatus* (N=1) were also genotyped for the more conserved large nuclear ribosomal (28S) gene fragment and their phylogenetic analyses (Figure 3) corroborated the among- *Waldo* relationships inferred from the mt 16S marker: (*Waldo digitatus* (*Waldo arthuri*, *Waldo paucitentaculatus*)). The mean genetic distances for this gene fragment were: 0.3% (*Waldo arthuri*/ *Waldo paucitentaculatus*), 2.2% (*Waldo digitatus* / *Waldo paucitentaculatus*), and 2.1% (*Waldo digitatus*/ *Waldo arthuri*). No intraspecific variations were detected for all three species.

## Discussion

The molecular phylogenetic and morphological data concur that the new species here described is a member of the genus *Waldo* and is sister to the closely related southwestern Atlantic *Waldo paucitentaculatus*. Although it is not uncommon for marine invertebrates to have sister taxa in different ocean basins, it is a little surprising in this case because all other records for this genus are in high latitude southern hemisphere locations and all studied congeners apparently also lack pelagic larval development (Zelaya and Iuarte 2002, 2013). The large majority of commensal galeommatoideans brood their young to a straight-hinged veliger stage and then undergo a prolonged free-swimming obligatory planktotrophic phase prior to metamorphosis. However, absence of pelagic larvae does not seem to constrain the geographic range of species of *Waldo*. *Waldo parasiticus* has a circum-Antarctic distribution (Zelaya and Iuarte 2002) and *Waldo arthuri* has attained an extensive geographic range (Vancouver Island to San Diego). It is conceivable that *Waldo arthuri* will also be found throughout the range of its host, *Brisaster latifrons*, which has a documented distribution from the Bering Sea, Alaska to the Galapagos Islands, Ecuador (Lissner and Hart 1996).

## Supplementary Material

XML Treatment for Waldo

XML Treatment for Waldo arthuri
